# Profile, follow-up adherence and progression of people with advanced chronic kidney disease in specialized care centers: a retrospective study, Santa Catarina state, Brazil, 2021-2024

**DOI:** 10.1590/S2237-96222026v35e20250690.en

**Published:** 2026-04-10

**Authors:** Fabiana Baggio Nerbass, Marina Abritta Hanauer, Isadora Felski da Silva, Thais Franz da Costa, Rafael Marques da Silva, Larissa Fabre, Marcos Alexandre Vieira, Viviane Calice-Silva

**Affiliations:** 1 Fundação Pró-Rim, Joinville, SC, Brasil; 2 Fundação Pró-Rim, Balneário Camboriú, SC, Brasil; 3 Centro de Tratamento de Doenças Renais, Jaraguá do Sul, SC, Brasil; 4 Centro de Tratamento de Doenças Renais, Mafra, SC, Brasil; 5 Centro de Tratamento de Doenças Renais, Joinville, SC, Brasil

**Keywords:** Chronic Kidney Disease, Epidemiology, Brazilian Unified Health System, Glomerular Filtration Rate, Retrospective Studies, Enfermedad Renal Crónica, Epidemiología, Sistema Único de Salud, Tasa de Filtración Glomerular, Estudios Retrospectivos

## Abstract

**Objectives:**

To characterize the profile of people with advanced chronic kidney disease treated in specialized outpatient centers and to describe adherence to follow-up and assess the impact of specialized follow-up by comparing the glomerular filtration rate at baseline and after one year of follow-up.

**Methods:**

This was a retrospective multicenter study that included people with stage 4 and 5 chronic kidney disease treated between November 2021 and October 2024, in five specialized outpatient centers in Santa Catarina state. The 12-month follow-up rate and changes in kidney function were assessed through the estimated glomerular filtration rate of people who started follow-up by October 2023.

**Results:**

Of the 547 people cared for during the study period, 83.0% were at stage 4 of the disease, 80.6% were elderly (>60 years), 91.8% had hypertension, 51.6% had diabetes and 65.6% were overweight or obese. Of the 237 people who started follow-up by October 2023, 211 (90.2%) completed one year of follow-up. The median estimated glomerular filtration rate remained stable during the period (22 [18-26] versus 22 [18-27] ml/min/1.73m2; p-value 0.300). Five people (2.1%) progressed to renal replacement therapy.

**Conclusion:**

The population served was predominantly elderly and presented multiple risk factors for progression of chronic kidney disease. The high follow-up rate over one year and the preservation of kidney function reinforced the importance of specialized follow-up in the care of people with advanced chronic kidney disease.

## Resumen

**Objetivos:**

Caracterizar el perfil de las personas con enfermedad renal crónica avanzada atendidas en centros ambulatorios especializados, describir la adherencia al seguimiento y evaluar el impacto del seguimiento especializado comparando la tasa de filtración glomerular al inicio y al año de seguimiento.

**Métodos:**

Estudio multicéntrico retrospectivo que incluyó a personas con enfermedad renal crónica en estadios 4 y 5 atendidas entre noviembre de 2021 y octubre de 2024 en cinco unidades ambulatorias especializadas del estado de Santa Catarina. La tasa de seguimiento a 12 meses y los cambios en la función renal se evaluaron mediante la tasa de filtración glomerular estimada de las personas que iniciaron el seguimiento hasta octubre de 2023.

**Resultados:**

De las 547 personas atendidas durante el estudio, el 83,0 % se encontraba en estadio 4 de la enfermedad, el 80,6 % eran personas mayores (>60 años), el 91,8 % presentaba hipertensión, el 51,6 % diabetes y el 65,6 % presentaba sobrepeso u obesidad. De las 237 personas que iniciaron el seguimiento en octubre de 2023, 211 (90,2 %) completaron un año de seguimiento. La mediana de la tasa de filtración glomerular estimada se mantuvo estable durante el período (22 [18-26] frente a 22 [18-27] ml/min/1,73 m2; valor p: 0,300). Cinco personas (2,1 %) progresaron a terapia de reemplazo renal.

**Conclusión:**

La población atendida fue predominantemente de edad avanzada y presentó múltiples factores de riesgo para la progresión de la enfermedad renal crónica. La alta tasa de seguimiento a un año y la preservación de la función renal reforzaron la importancia del seguimiento especializado en la atención de personas con enfermedad renal crónica avanzada.

**Ethical aspects**

This research respected ethical principles, having obtained the following approval data::

Research ethics committee: Hospital Regional Hans Dieter Schimidt

Opinion number: 7,623,770

Approval date: 6/6/2025

Certificate of submission for ethical appraisal: 89041325.9.1001.5363

Informed consent form: Dismissed.

## Introduction 

Chronic kidney disease is a growing public health problem and, in 2023, it ranked seventh among the leading risk factors for global mortality (1). This disease is diagnosed by assessing the estimated glomerular filtration rate and the albumin/creatinine ratio in a single urine sample, which is also recognized as a marker of its progression (2).

In Brazil, the estimated prevalence of chronic kidney disease was approximately 7% among adults and 21% among the elderly in 2015 (3). However, these numbers may be underestimated, since the urinary albumin/creatinine ratio was not assessed (2). In a randomly selected urban cohort from the Southern region of Brazil, composed of more than 5,000 adults, 11% were identified as having chronic kidney disease in 2017 (4). This percentage was similar to that observed in the analysis that assessed kidney function tests in 4.5 million individuals attended in clinical laboratories throughout the country, between 2018 and 2021, registering 11% prevalence (5).

As chronic kidney disease progresses to more severe stages, the impacts on people’s well-being and quality of life become more evident (6). Furthermore, disease progression is linked to increased social and healthcare costs (7). Implementing a pre-dialysis care program has been shown to reduce average healthcare costs by US$8,508.26 ± US$432.02 per year of avoided dialysis treatment (8). Therefore, attending appointments regularly and adhering to treatment are essential actions for maximizing the benefits of specialized follow-up (9,10).

Santa Catarina, a state in the Southern region of Brazil and the tenth most populous in the country, has the highest life expectancy among the country’s Federative Units. In 2023, it reached 80.9 years, while the national average was 77.4 years (11). Population aging and the increase in traditional risk factors for chronic kidney disease, such as hypertension, diabetes and cardiovascular diseases, signal a worrying scenario and highlight the urgent need for action by governments and the health sector (12).

In response to this reality, the government of Santa Catarina implemented the Care Pathway for People with Chronic Kidney Disease policy in November 2021. Care pathways define the care flows that must be guaranteed to users of the Brazilian Unified Health System (*Sistema Único de Saúde*, SUS), aiming to meet their health needs. Among the measures established, the state-level increase in renal replacement therapy stands out, aimed at outpatient care facilities specialized in chronic kidney disease, with the requirement to make appointments available for state appointment allocation. It focuses especially on people who are at stages 4 and 5, when the glomerular filtration rate is between 15 and 29 ml/min/1.73m2 and below 15 ml/min/1.73m2, respectively (13).

Given that this is a recent policy and the scarcity of data on the impact of regional public policies aimed at the care of advanced chronic kidney disease, understanding the profile of the people served, their adherence rate to follow-up, and their potential clinical outcomes is essential for assessing effectiveness and supporting improvement of care strategies.

The objectives of this study were to describe the profile of people with stage 4 and 5 chronic kidney disease treated in five specialized outpatient care services in Santa Catarina, to determine the proportion of people who completed one year of follow-up, and to assess the impact of specialized follow-up by comparing the glomerular filtration rate at baseline and after one year of follow-up.

## Methods 

### Study design and setting 

This is a retrospective study that included adults treated between November 2021 and October 2024 at five specialized chronic kidney disease outpatient care centers. These centers were located in four different cities in the following regions of Santa Catarina: *Foz do Rio Itajaí* (Balneário Camboriú), *Planalto Norte* (Mafra) and Nordeste (Joinville and Jaraguá do Sul). 

The centers, two philanthropic and three private, are partners and share the same electronic medical record system. These centers are the only ones in each city to be integrated into the state’s care pathway program. The people referred to the specialized services already had diagnosis of chronic kidney disease and were having SUS follow-up in their respective municipalities.

### Participants 

We assessed all adults followed up at the participating institutions and included in the state care pathway program who presented, at the first consultation, an estimated glomerular filtration rate using the CKD-EPI 2021 formula (14) indicative of stage 4 chronic kidney disease (15-29 ml/min/1.73m^2^) or non-dialysis stage 5 (<15 ml/min/1.73m^2^). Those with an estimated glomerular filtration rate above 29 ml/min/1.73m^2^ were excluded.

### Variables 

Between October and December 2024, demographic data (sex, age, education level, race/skin color and municipality of residence) input to the system by administrative assistants were extracted from electronic medical records. Clinical variables included previous diagnoses of diabetes and hypertension recorded by physicians at the first consultation. Weight and height data, measured or self-reported by individuals, were also collected and used to calculate body mass index. The estimated glomerular filtration rate (from serum creatinine) was also recorded at the first medical assessment.

In order to compare kidney function after 12 months of follow-up, individuals with records containing estimated glomerular filtration rate information between 10 and 14 months after the first consultation were included. This interval was adopted to account for small variations in the scheduling of routine consultations and examinations, ensuring greater comprehensiveness and consistency in the analysis. The follow-up rate was also calculated, as well as the percentages of individuals who: did not return after the first consultation; did not complete 12 months of follow-up; and were discharged due to recovery of kidney function.

The participants were asked whether they had prior follow-up with a nephrologist, in order to assess the impact of specialized care. If so, they were asked whether it was at the same institution or at another. One of the participating units (Balneário Camboriú) already provided this service to the municipal health department before implementation of the state policy.

After the initial consultation, the frequency of each person’s return visits was determined by the attending nephrologist, taking into account kidney function and clinical condition, with intervals ranging from one week to three months.

The participating outpatient centers used the Ministry of Health Care for Patients with Chronic Kidney Disease clinical guidelines and the Care Pathway for People with Chronic Kidney Disease in Santa Catarina as protocols for case management (13,15). The participating centers used the same electronic system (software) for clinical records, which ensured homogeneity in information collection and storage.

### Statistical methods 

Statistical analysis was performed using IBM SPSS Statistics, version 21.0 (IBM Corp., Armonk, New York, United States). The results were presented as percentage, mean and standard deviation or median and interquartile range, according to the distribution of the variables. Data normality was verified using the Kolmogorov-Smirnov and Shapiro-Wilk tests. The Wilcoxon signed-rank test was used to compare the estimated initial and final glomerular filtration rate. P-values<0.050 were taken to be statistically significant.

## Results 

Between November 2021 and October 2024, 547 people with stage 4 and 5 chronic kidney disease received care and were included in the study (age=70.6±13.5 years; body mass index=27.6±5.2 kg/m^2^; estimated glomerular filtration rate=22 [17-26 ml/min/1.73m^2^]). 233 patients were seen at the Balneário Camboriú unit; 180 at the two Joinville units; 87 at Jaraguá do Sul; and 47 at Mafra.

54.8% were female, 80.6% were elderly, 58.9% did not have complete elementary education and 47.9% lived in a municipality neighboring the care unit. More than half (51.6%) had diabetes, and two-thirds had nutritional diagnosis of overweight or obesity. At the start of follow-up, 82.8% were at stage 4 of the disease (Table 1).

**Table 1:** Characteristics of the people cared between November 2021 and October 2024 in participating outpatient centers. Santa Catarina, 2021-2024 (n=547)

Characteristics	n (%)
Sex	
Female	300 (54.8)
Male	247 (45.2)
**Age** (years)	
20-39	16 (2.9)
40-59	90 (16.5)
60-79	294 (53.7)
≥80	147 (26.9)
**Race/skin color**	
White	473 (86.5)
Mixed race	39 (7.1)
Black	26 (4.7)
Asian	9 (1.6)
**Schooling** (n=416)	
Illiterate	33 (7.9)
Incomplete elementary	212 (51.0)
Complete elementary	78 (18.7)
High school or higher education	93 (22.3)
**Place of residence**	
Municipality with outpatient center	285 (52.1)
Neighboring municipality	261 (47.9)
Comorbidities	
Diabetes	282 (51.6)
Hypertension	502 (91.8)
**Nutritional status** (n=498)	
Underweight	9 (1.8)
Normal weight	162 (32.5)
Overweight	191 (38.3)
Obesity	136 (27.3)
**Estimated glomerular filtration rate** (ml/min/1.73m^2^)	
15-29 (stage 4)	453 (82.8)
<15 (stage 5)	94 (17.2)

**Figure 1:**
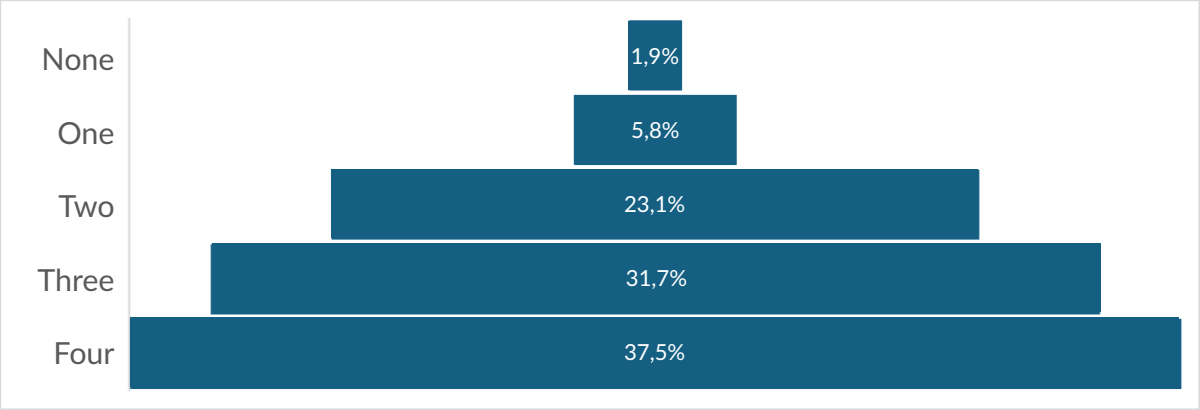
Patient distribution per number of chronic kidney disease risk factors (age ≥ 60 years, diabetes, hypertension and overweight/obesity). Santa Catarina, 2021-2024 (n = 547)

Distribution of individuals according to the number of predetermined risk factors for the progression of chronic kidney disease is shown (Figure 1). Diabetes, hypertension, advanced age and overweight or obesity were the factors assessed. More than two-thirds presented three or all four risk factors.

Of the total number of people treated, 237 began follow-up by October 2023, thus enabling them to be monitored for at least 12 months. Among them, 64 (27%) had previously been seen by a nephrologist, 24 at the center itself and 40 at other services. Of the 237 people, 3 were discharged due to recovery of kidney function. Among the remaining 234, 6 (2.6%) did not return after the first consultation, and 18 did not complete one year of follow-up (7.7%) for unknown reasons.

**Figure 2:**
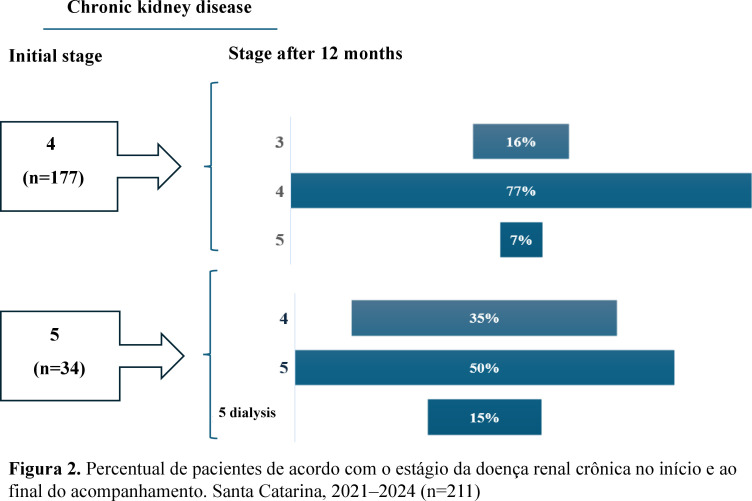
Percentage of patients according to chronic kidney disease stage at the beginning and end of follow-up. Santa Catarina, 2021-2024 (n=211)

The results regarding kidney function and chronic kidney disease stage of the 211 individuals (90.2%) who completed one year of follow-up are shown (Table 2; Figure 2). The median estimated glomerular filtration rate remained stable throughout the period in the entire sample and when compared according to prior follow-up or no prior follow-up with a nephrologist (data not shown). The median estimated glomerular filtration rate also did not vary after one year in relation to individuals who started at stage 4. In total, 16.0% of these individuals returned to stage 3B (>29 ml/min/1.73m^2^), and 7.0% progressed to stage 5.

**Table 2:** Median and interquartile ranges (IQR) of the estimated glomerular filtration rate at the start and after one year of follow-up, according to chronic kidney disease stage. Santa Catarina, 2021-2024 (n=206)

Initial stage	Initial estimated glomerular filtration rate (ml/min/1.73m^2^) Median (IQR)	Final estimated glomerular filtration rate (ml/min/1.73m^2^) Median (IQR)	p-value
Stages 4 and 5^a^	22 (18-26)	22 (17-26)	0.300
Stage 4 (n=177)	23 (20-26)	23 (18-27)	0.860
Stage 5^a^ (n=29)	11 (9-14)	12 (9-19)	0.020

^a^The five people who progressed to renal replacement therapy were excluded; Statistical test: Wilcoxon signed-rank test

Among those who began follow-up at stage 5, 35.0% showed improvement in the estimated glomerular filtration rate, returning to stage 4, and 15.0% progressed to requiring renal replacement therapy (stage 5D). Excluding those in stage 5D, there was a slight but statistically significant improvement in kidney function.

## Discussion 

This multicenter study demonstrated that the profile of individuals with stage 4 and 5 chronic kidney disease treated at specialized outpatient centers was predominantly elderly with multiple risk factors. A high follow-up rate was observed in the participating services over 12 months, and kidney function levels were maintained during this period.

The higher proportion of females in this sample of people with advanced chronic kidney disease was consistent with national and international findings (3,16). In addition to higher life expectancy among females (in Santa Catarina, they live on average 6.4 years longer than men) (11), biological, cultural and behavioral factors, as well as their interactions, have been proposed to explain the sex disparities observed globally (17).

Average age over 70 years, with more than a quarter aged 80 or older, reflected the high prevalence of the disease as age advances. In addition to the natural loss of kidney function resulting from physiological changes, greater exposure to comorbidities associated with kidney damage (hypertension and diabetes), as well as the onset of other conditions common in this age group (cardiovascular diseases, benign prostatic hyperplasia and cancer) may contribute to this finding (18). A North American study reported average age close to 80 years in the most advanced stages of the disease (19). We did not identify directly comparable national studies.

The educational level of this sample, in which approximately 8% were illiterate and 41% had only completed elementary education, was lower than that of the general population of Santa Catarina, for whom the prevalence rates in 2022 were 3% and 54%, respectively (20). This difference can be explained, in part, by the age profile of the sample, composed predominantly of elderly people who had less access to formal education in childhood compared to the current adult population. Association between lower education levels and higher prevalence of chronic kidney disease has already been described in the Brazilian medical literature and contributes to explaining this result (21).

Hypertension and diabetes are the two main causes of chronic kidney disease globally (2) and, as in this sample, hypertension is the most common comorbidity in other cohorts (22). In addition to being a primary factor for kidney damage, its prevalence increases as kidney function declines, given the central role of the kidneys in blood pressure control (23). More than half of the participants had diabetes, the nephropathy of which is related to metabolic, hemodynamic and cellular mechanisms associated with chronic hyperglycemia (24).

More than one-third of the participants were classified as overweight (38%), and more than a quarter as obese (27%). Obesity is a significant risk factor for the development and progression of the disease, through mechanisms that include hemodynamic changes, inflammation, oxidative stress and activation of the renin-angiotensin-aldosterone system (25). Conditions associated with obesity, particularly diabetes and hypertension, can coexist and worsen kidney damage (26). Coexistence of hypertension, diabetes, obesity and advanced age was prevalent in the sample, with almost 70% of participants presenting at least three of these four conditions (Figure 1).

Ninety percent of the participants had a return visit between 10 and 14 months after their first consultation, which indicated a low rate of absenteeism or dropout. National studies on absenteeism in specialized SUS consultations reported considerably higher rates than those observed in this study (27,28). In those investigations, absenteeism was influenced by the waiting time until the appointment (27) and explained mainly by lack of transport, financial difficulties and forgetting (28).

Several factors may have contributed to the positive outcome, for example, the short waiting time for the first consultation (less than 30 days in most cases) and the immediate scheduling of the follow-up appointment. In addition, people from neighboring municipalities had access to free transportation provided by local health departments. On an individual level, the advanced stage of the disease, the presence of multiple comorbidities, and participant satisfaction with the care received may have been determining factors.

Kidney function preservation as well as the low rate of people who progressed to renal replacement therapy over one year of follow-up suggest potentially effective therapeutic management in this population. Although the lack of detailed information on clinical factors and interventions limits further analysis, it is plausible to suggest that regular follow-up contributed to these outcomes, as it allows for therapeutic adjustments to control risk factors better, coupled with the introduction of contemporary strategies to protect the kidneys (2).

This study added relevant information to the national scenario by describing, for the first time, the profile of people with stage 4 and 5 chronic kidney disease treated in specialized centers linked to the SUS in Santa Catarina. High adherence rates to outpatient follow-up and maintenance of kidney function over a one-year period were observed, these being results rarely reported in the national clinical literature. These findings suggest that the organization of regional care pathways, with strategies focused on timely access and continuous follow-up, can contribute to reducing the rate of disease progression and improving SUS sustainability.

Among the limitations of this study, the retrospective nature of the analysis and the absence of a control group stood out, these being factors that restrict the possibility of establishing causal relationships. Also noteworthy were the lack of analysis of the therapeutic approach, the use of self-reported data, and the lack of information for a more detailed characterization of the sample regarding comorbidities and risk factors. The study’s strength lies in its multicenter nature and its relevance in addressing a topic that is still little explored in the national literature.

The conclusion was reached that the population served in the first three years of implementation of the Care Pathway for People with Chronic Kidney Disease in Santa Catarina, across five specialized outpatient centers, was predominantly female, elderly and presented multiple risk factors. The high follow-up rate over one year and maintenance of kidney function in these individuals highlighted the relevance of specialized follow-up in the care of people with advanced chronic kidney disease.

Although it is not possible to establish causality, these findings suggest that this type of care may be associated with better long-term outcomes for individuals and reduced costs for healthcare systems. Furthermore, the importance of strengthening organizational strategies within the SUS that facilitate access and adherence to specialized care was highlighted, such as quicker scheduling of initial appointments, immediate scheduling of follow-up visits, and provision of free transportation. This contributes to slowing the progression of the disease and promoting greater sustainability of the system.

## Data Availability

The data used in this study are available, upon request to the corresponding author, for peer reviewers and readers who may be interested.
