# Supplementary material for: Profile, follow-up adherence and progression of people with advanced chronic kidney disease in specialized care centers: a retrospective study, Santa Catarina state, Brazil, 2021-2024
Source: Epidemiol Serv Saude. 2026 Apr 10;35:e20250690. doi: 10.1590/S2237-96222026v35e20250690.en (PMC13078772; doi:10.1590/S2237-96222026v35e20250690.en)
Supplement: Translation

# Translation: Perfil, adesão ao seguimento e evolução de pessoas com doença renal crônica avançada em centros especializados: estudo retrospectivo, Santa Catarina, 2021-2024

**DOI:** 10.1590/S2237-96222026v35e20250690.pt

**Authors:** Fabiana Baggio Nerbass, Marina Abritta Hanauer, Isadora Felski da Silva, Thais Franz da Costa, Rafael Marques da Silva, Larissa Fabre, Marcos Alexandre Vieira, Viviane Calice-Silva

**Affiliations:** 1 Fundação Pró-Rim, Joinville, SC, Brasil; 2 Fundação Pró-Rim, Balneário Camboriú, SC, Brasil; 3 Centro de Tratamento de Doenças Renais, Jaraguá do Sul, SC, Brasil; 4 Centro de Tratamento de Doenças Renais, Mafra, SC, Brasil; 5 Centro de Tratamento de Doenças Renais, Joinville, SC, Brasil

**Keywords:** Insuficiência Renal Crônica, Epidemiologia, Sistema Único de Saúde, Taxa de Filtração Glomerular, Estudos Retrospectivos

## Abstract

**Objetivos:**

Caracterizar o perfil das pessoas com doença renal crônica avançada atendidas em centros ambulatoriais especializados e descrever a adesão ao acompanhamento e avaliar o impacto e avaliar o impacto do acompanhamento especializado comparando-se a taxa de filtração glomerular no início e após um ano de acompanhamento.

**Métodos:**

Estudo multicêntrico retrospectivo que incluiu pessoas com doença renal crônica nos estágios 4 e 5 atendidas entre novembro de 2021 e outubro de 2024, em cinco unidades ambulatoriais especializadas de Santa Catarina. Foram avaliadas a taxa de acompanhamento em 12 meses e as mudanças na função renal por meio da taxa de filtração glomerular estimada das pessoas que iniciaram o acompanhamento até outubro de 2023.

**Resultados:**

Das 547 pessoas atendidas no período em estudo, 83,0% estavam no estágio 4 da doença, 80,6% eram idosos (>60 anos), 91,8% tinham hipertensão, 51,6% tinham diabetes e 65,6% apresentavam sobrepeso ou obesidade. Das 237 pessoas que iniciaram o acompanhamento até outubro de 2023, 211 (90,2%) completaram um ano de seguimento. A mediana da taxa de filtração glomerular estimada se manteve estável no período (22 [18-26] versus 22 [18-27] ml/min/1,73m2; p-valor 0,300). Cinco pessoas (2,1%) evoluíram para a terapia renal substitutiva.

**Conclusão:**

A população atendida era predominantemente idosa e apresentava múltiplos fatores de risco para a progressão da doença renal crônica. A elevada taxa de acompanhamento ao longo de um ano e a preservação da função renal reforçaram a importância do seguimento especializado na atenção às pessoas com doença renal crônica avançada.

**Aspectos éticos**

Esta pesquisa respeitou os princípios éticos, obtendo os seguintes dados de aprovação:

Comitê de ética em pesquisa: Hospital Regional Hans Dieter Schimidt

Número do parecer: 7.623.770

Data de aprovação: 6/6/2025

Certificado de apresentação de apreciação ética: 89041325.9.1001.5363

Registro do consentimento livre e esclarecido: Dispensado.

## Introdução 

A doença renal crônica é um problema de saúde pública crescente e, em 2023, ocupava a sétima posição entre os principais fatores de risco para mortalidade global (1). Essa doença é diagnosticada por meio da avaliação da taxa de filtração glomerular estimada e da relação albumina/creatinina em amostra de urina isolada, que também é reconhecida como marcador de sua progressão (2). 

No Brasil, a prevalência estimada de doença renal crônica foi de aproximadamente 7% entre adultos e 21% entre os idosos em 2015 (3). No entanto, esses números podem estar subestimados, uma vez que a relação albumina/creatinina urinária não foi avaliada (2). Em uma coorte urbana da região Sul do Brasil, selecionada aleatoriamente, composta de mais de 5 mil adultos, 11% foram identificados com doença renal crônica em 2017 (4). Esse percentual foi semelhante ao observado na análise que avaliou exames de função renal em 4,5 milhões de indivíduos atendidos em laboratórios clínicos de todo o país, entre 2018 e 2021, registrando-se prevalência de 11% (5).

À medida que a doença renal crônica avança para os estágios mais graves, os impactos sobre o bem-estar e a qualidade de vida das pessoas se tornam mais evidentes (6). Além disso, a progressão da doença está relacionada ao aumento dos custos sociais e assistenciais (7). Foi demonstrado que a implementação de um programa de atendimento pré-dialítico pode reduzir os custos médios de saúde em US$ 8.508,26±US$ 432,02 por ano de tratamento dialítico evitado (8). Para tanto, comparecer frequentemente às consultas e aderir ao tratamento são ações essenciais para potencializar os benefícios do acompanhamento especializado (9,10). 

Santa Catarina, estado da região Sul do Brasil e o décimo mais populoso do país, conta com a maior expectativa de vida entre as Unidades da Federação. Em 2023, alcançou-se a marca de 80,9 anos, enquanto a média nacional era 77,4 anos (11). O envelhecimento populacional e o aumento dos fatores de risco tradicionais para a doença renal crônica, como hipertensão, diabetes e doenças cardiovasculares, sinalizam um cenário preocupante e ressaltam a urgente necessidade de ações por parte dos governos e do setor de saúde (12).

Em resposta a essa realidade, o governo de Santa Catarina implementou a política da Linha de Cuidado da Pessoa com Doença Renal Crônica em novembro de 2021. As linhas de cuidado definem os fluxos assistenciais que devem ser garantidos aos usuários do Sistema Único de Saúde (SUS), visando atender às necessidades de saúde destes. Entre as medidas estabelecidas, destaca-se o incremento estadual na terapia renal substitutiva destinado às unidades de atenção ambulatorial especializadas em doença renal crônica, com a exigência de disponibilizarem agenda para a regulação estadual. Foca-se especialmente as pessoas que se encontram nos estágios 4 e 5, quando a taxa de filtração glomerular estiver entre 15 e 29 ml/min/1,73m^2^ e abaixo de 15 ml/min/1,73m^2^, respectivamente (13).

Por se tratar de uma política recente e diante da escassez de dados sobre o impacto de políticas públicas regionais voltadas ao cuidado da doença renal crônica avançada, conhecer o perfil das pessoas atendidas, sua taxa de adesão ao acompanhamento e seus potenciais desfechos clínicos são essenciais para avaliar a efetividade e subsidiar o aprimoramento das estratégias de cuidado.

Os objetivos deste estudo foram descrever o perfil das pessoas com doença renal crônica nos estágios 4 e 5 atendidas em cinco serviços catarinenses de atenção ambulatorial especializada, determinar a proporção de pessoas que completaram um ano de seguimento e avaliar o impacto do acompanhamento especializado comparando-se a taxa de filtração glomerular no início e após um ano de acompanhamento.

## Métodos 

### Desenho do estudo e contexto

Trata-se de estudo retrospectivo que incluiu pessoas adultas atendidas entre novembro de 2021 e outubro de 2024 em cinco unidades de atenção ambulatorial especializadas em doença renal crônica. Essas unidades estavam localizadas em quatro diferentes cidades das seguintes regiões de Santa Catarina: Foz do Rio Itajaí (Balneário Camboriú), Planalto Norte (Mafra) e Nordeste (Joinville e Jaraguá do Sul). 

As unidades, duas filantrópicas e três privadas, são parceiras e compartilham o mesmo sistema de prontuários eletrônicos. Tais unidades são as únicas de cada cidade a integrar o programa da linha de cuidado estadual. As pessoas encaminhadas aos serviços especializados já tinham diagnóstico de doença renal crônica e eram acompanhadas no SUS de seus respectivos municípios.

### Participantes 

Foram avaliadas todas as pessoas adultas acompanhadas nas instituições participantes e incluídas no programa da linha de cuidado estadual que apresentaram, na primeira consulta, taxa de filtração glomerular estimada pela fórmula CKD-EPI 2021 (14) indicativa de doença renal crônica no estágio 4 (15-29 ml/min/1,73m^2^) ou no estágio 5 não dialítico (<15 ml/min/1,73m^2^). Aquelas com taxa de filtração glomerular estimada acima de 29 ml/min/1,73m^2^ foram excluídas. 

### Variáveis 

Entre outubro e dezembro de 2024, dados demográficos (sexo, idade, grau de instrução, raça/cor da pele e município de residência) inseridos no sistema pelos auxiliares administrativos foram extraídos dos prontuários eletrônicos. As variáveis clínicas incluíram diagnóstico prévio de diabetes e hipertensão registrados pelos médicos na primeira consulta. Ainda foram coletados dados de peso e altura mensurados ou autorreferidos pelas pessoas, utilizados para cálculo do índice de massa corporal. A taxa de filtração glomerular estimada (a partir da creatinina sérica) foi igualmente registrada na primeira avaliação médica.

Para comparar a função renal após 12 meses de acompanhamento, foram incluídas as pessoas que apresentavam registro contendo a informação da taxa de filtração glomerular estimada entre 10 e 14 meses após a primeira consulta. Esse intervalo foi adotado para contemplar pequenas variações no agendamento de consultas e exames de rotina, o que garantiu maior abrangência e consistência na análise. A taxa de acompanhamento também foi calculada, assim como os percentuais de pessoas que: não retornaram após a primeira consulta; não completaram 12 meses de seguimento; e receberam alta por recuperação da função renal. 

Foi perguntado às pessoas se havia acompanhamento prévio com nefrologista, com o objetivo de avaliar o impacto da atenção especializada. Em caso afirmativo, perguntava-se se na mesma instituição ou em outra. Uma das unidades participantes (Balneário Camboriú) já prestava esse serviço à secretaria de saúde do município antes da implementação da política estadual.

Após a primeira consulta, a frequência de retorno de cada pessoa era definida pelo nefrologista responsável, considerando a função renal e a condição clínica, com intervalos que variavam de uma semana a três meses.

Os centros participantes utilizaram como protocolos para manejo das pessoas as diretrizes estabelecidas pelo Cuidado ao Paciente com Doença Renal Crônica do Ministério da Saúde e pela Linha de Cuidado da Pessoa com Doença Renal Crônica em Santa Catarina (13,15). As unidades participantes utilizaram o mesmo sistema eletrônico (software) para o registro clínico, o que assegurou homogeneidade na coleta e no armazenamento das informações.

### Métodos estatísticos 

A análise estatística foi realizada no IBM SPSS Statistics, versão 21.0 (IBM Corp., Armonk, New York, Estados Unidos). Os resultados foram apresentados em percentual, média e desvio-padrão ou mediana e interquartis, conforme a distribuição das variáveis. A normalidade dos dados foi verificada pelos testes de Kolmogorov-Smirnov e Shapiro-Wilk. O teste de postos sinalizados de Wilcoxon foi utilizado para comparar a taxa de filtração glomerular estimada inicial e final. A significância estatística foi considerada para p-valor<0,050.

## Resultados 

Entre novembro de 2021 e outubro de 2024, 547 pessoas nos estágios 4 e 5 da doença renal crônica foram atendidas e incluídas no estudo (idade=70,6±13,5 anos; índice de massa corporal=27,6±5,2 kg/m^2^; taxa de filtração glomerular estimada=22 [17-26 ml/min/1,73m^2^]). Ocorreram 233 atendimentos na unidade de Balneário Camboriú; 180 eram provenientes das duas unidades de Joinville; 87, de Jaraguá do Sul; e 47, de Mafra. 

Destes, 54,8% eram do sexo feminino, 80,6% eram idosos, 58,9% não tinham o ensino fundamental completo e 47,9% residiam em município vizinho à unidade de atendimento. Mais da metade (51,6%) tinha diabetes, e dois terços tinham o diagnóstico nutricional de sobrepeso ou obesidade. No início do acompanhamento, 82,8% estavam no estágio 4 da doença (Tabela 1).

**Tabela 1:** Características das pessoas atendidas entre novembro de 2021 e outubro de 2024 nas unidades participantes. Santa Catarina, 2021-2024 (n=547)

Características	n (%)
Sexo	
Feminino	300 (54,8)
Masculino	247 (45,2)
**Idade** (anos)	
20-39	16 (2,9)
40-59	90 (16,5)
60-79	294 (53,7)
≥80	147 (26,9)
**Raça/cor da pele**	
Branca	473 (86,5)
Parda	39 (7,1)
Preta	26 (4,7)
Amarela	9 (1,6)
**Grau de instrução** (n=416)	
Não alfabetizado	33 (7,9)
Fundamental incompleto	212 (51,0)
Fundamental completo	78 (18,7)
Ensino médio ou superior	93 (22,3)
Residência	
Município com unidade de atendimento	285 (52,1)
Município vizinho	261 (47,9)
Comorbidades	
Diabetes	282 (51,6)
Hipertensão	502 (91,8)
**Estado nutricional** (n=498)	
Baixo peso	9 (1,8)
Eutrofia	162 (32,5)
Sobrepeso	191 (38,3)
Obesidade	136 (27,3)
**Taxa de filtração glomerular estimada** (ml/min/1,73m^2^)	
15-29 (estágio 4)	453 (82,8)
<15 (estágio 5)	94 (17,2)

Apresentou-se a distribuição das pessoas de acordo com a quantidade de fatores de risco predeterminados para a progressão da doença renal crônica (Figura 1). Diabetes, hipertensão, idade avançada e sobrepeso ou obesidade foram os fatores avaliados. Mais de dois terços apresentavam três ou todos os quatro fatores de risco. 

**Figura 1:** Distribuição dos pacientes segundo o número de fatores de risco para doença renal crônica (idade ≥ 60 anos, diabetes, hipertensão e sobrepeso/obesidade). Santa Catarina, 2021–2024 (n = 547).

Do total de pessoas atendidas, 237 iniciaram o acompanhamento até outubro de 2023, o que possibilitou que fossem monitoradas por pelo menos 12 meses. Entre elas, 64 (27%) eram atendidas previamente por nefrologista, sendo 24 na própria unidade e 40 em outros serviços. Das 237 pessoas, 3 receberam alta por recuperação da função renal. Entre as 234 restantes, 6 (2,6%) não retornaram após a primeira consulta, e 18 não completaram um ano de seguimento (7,7%) por motivos desconhecidos. 

Apresentaram-se os resultados referentes à função renal e ao estágio da doença renal crônica das 211 pessoas (90,2%) que completaram um ano de acompanhamento (Tabela 2; Figura 2). A mediana da taxa de filtração glomerular estimada se manteve estável ao longo do período em toda a amostra e quando comparada por seguimento prévio ou não com nefrologista (dados não mostrados). A mediana da taxa de filtração glomerular estimada também não variou após um ano com relação às pessoas que iniciaram no estágio 4. No total, 16,0% dessas pessoas regrediram para o estágio 3B (>29 ml/min/1,73m^2^), e 7,0% evoluíram para o estágio 5. 

**Tabela 2:** Mediana e intervalos interquartis (IIQ) da taxa de filtração glomerular estimada no início e após um ano de acompanhamento, por estágio da doença renal crônica. Santa Catarina, 2021-2024 (n=206)

Estágio inicial	Taxa de filtração glomerular estimada inicial (ml/min/1,73m^2^) Mediana (IIQ)	Taxa de filtração glomerular estimada final (ml/min/1,73m^2^) Mediana (IIQ)	p-valor
Estágios 4 e 5^a^	22 (18-26)	22 (17-26)	0,300
Estágio 4 (n=177)	23 (20-26)	23 (18-27)	0,860
Estágio 5^a^ (n=29)	11 (9-14)	12 (9-19)	0,020

^a^As cinco pessoas que evoluíram para terapia renal substitutiva foram excluídas. Teste estatístico: Teste de postos sinalizados de Wilcoxon

**Figura 2:** Percentual de pacientes de acordo com o estágio da doença renal crônica no início e ao final do acompanhamento. Santa Catarina, 2021–2024 (n=211)

Entre as pessoas que iniciaram o acompanhamento já no estágio 5, 35,0% apresentaram melhora da taxa de filtração glomerular estimada retornando ao estágio 4, e 15,0% evoluíram com necessidade de terapia renal substitutiva (estágio 5D). Excluídas aquelas em estágio 5D, houve melhora discreta mas estatisticamente significante da função renal.

## Discussão 

Neste estudo multicêntrico, demonstrou-se que o perfil das pessoas com doença renal crônica nos estágios 4 e 5 atendidas em centros ambulatoriais especializados foi predominantemente de idosos com múltiplos fatores de risco. Observaram-se a elevada taxa de acompanhamento nos serviços participantes ao longo de 12 meses e a manutenção dos níveis de função renal das pessoas durante esse período.

A maior proporção do sexo feminino nesta amostra de pessoas com doença renal crônica avançada foi consistente com achados nacionais e internacionais (3,16). Além da maior expectativa de vida em pessoas do sexo feminino (em Santa Catarina, elas vivem em média 6,4 anos a mais do que os homens) (11), fatores biológicos, culturais, comportamentais, bem como suas interações, foram propostos para explicar as disparidades de sexo observadas globalmente (17).

A média de idade superior a 70 anos, com mais de um quarto das pessoas com 80 anos ou mais, refletiu a alta prevalência da doença com o avançar da idade. Além da perda natural da função renal decorrente de mudanças fisiológicas, a maior exposição a comorbidades associadas ao dano renal (hipertensão e diabetes), bem como o surgimento de outras condições comuns nessa faixa etária (doenças cardiovasculares, hiperplasia prostática e câncer) podem contribuir para esse achado (18). Um estudo norte-americano relatou uma média de idade próxima de 80 anos nos estágios mais avançados da enfermidade (19). Não identificamos estudos nacionais diretamente comparáveis.

O nível de escolaridade desta amostra, em que aproximadamente 8,0% eram analfabetos e 41,0% tinham apenas o ensino fundamental completo, foi inferior ao da população geral de Santa Catarina, cujas prevalências, em 2022, eram de 3% e 54%, respectivamente (20). Essa diferença pode ser explicada, em parte, pelo perfil etário da amostra, composta predominantemente de idosos que tiveram menor acesso à educação formal na infância em comparação com a população adulta atual. A associação entre menor escolaridade e maior prevalência de doença renal crônica já foi descrita na literatura médica brasileira e contribui para justificar tal resultado (21).

Hipertensão e diabetes são as duas principais causas da doença renal crônica no mundo (2) e, assim como nesta amostra, a hipertensão é a comorbidade mais comum em outras coortes (22). Além de ser fator primário para o dano renal, sua prevalência aumenta conforme a função renal declina, dado o papel central dos rins no controle da pressão arterial (23). Mais da metade das pessoas apresentava diabetes, cuja nefropatia está relacionada a mecanismos metabólicos, hemodinâmicos e celulares associados à hiperglicemia crônica (24).

Mais de um terço das pessoas foram classificadas com sobrepeso (38%), e mais de um quarto, com obesidade (27%). A obesidade é um fator de risco significativo para o desenvolvimento e a progressão da enfermidade, por mecanismos que incluem alterações hemodinâmicas, inflamação, estresse oxidativo e ativação do sistema renina-angiotensina-aldosterona (25). Condições associadas à obesidade, em particular diabetes e hipertensão, podem coexistir e agravar o dano renal (26). A coexistência de hipertensão, diabetes, obesidade e idade avançada foi prevalente na amostra, com quase 70% das pessoas apresentando pelo menos três dessas quatro condições (Figura 1).

Noventa por cento das pessoas tiveram registro de retorno entre 10 e 14 meses após a primeira consulta, o que indicou baixa taxa de absenteísmo ou abandono. Estudos nacionais sobre absenteísmo em consultas especializadas do SUS reportaram taxas consideravelmente superiores às observadas neste estudo (27,28). Nessas investigações, o absenteísmo foi influenciado pelo tempo de espera até a consulta (27) e justificado principalmente por falta de transporte, dificuldades financeiras e esquecimento (28). 

Vários fatores podem ter contribuído para o resultado positivo, por exemplo o curto tempo de espera para a primeira consulta (inferior a 30 dias na maioria dos casos) e o agendamento imediato do retorno. Além disso, as pessoas de municípios vizinhos contavam com transporte gratuito oferecido pelas secretarias de saúde locais. No aspecto individual, o estágio avançado da doença, a presença de múltiplas comorbidades e a satisfação das pessoas com o atendimento podem ter sido fatores determinantes.

A preservação da função renal e a baixa taxa de pessoas que progrediram para terapia renal substitutiva ao longo de um ano de seguimento sugerem o manejo terapêutico potencialmente eficaz nessa população. Embora a ausência de informações detalhadas sobre fatores clínicos e intervenções limite a análise mais aprofundada, é plausível apontar que o acompanhamento regular tenha contribuído para esses desfechos, em razão de possibilitar ajustes terapêuticos para melhor controle dos fatores de risco, aliado à introdução de estratégias contemporâneas para proteger os rins (2).

Este estudo acrescentou informações relevantes ao cenário nacional ao descrever, de forma inédita, o perfil de pessoas com doença renal crônica nos estágios 4 e 5 atendidas em centros especializados vinculados ao SUS em Santa Catarina. Observaram-se a elevada taxa de adesão ao acompanhamento ambulatorial e a manutenção da função renal ao longo de um ano, resultados pouco relatados na literatura clínica nacional. Esses achados sugeriram que a organização de linhas de cuidado regionais, com estratégias voltadas para o acesso oportuno e o seguimento contínuo, pode contribuir para reduzir a velocidade de progressão da doença e melhorar a sustentabilidade do SUS.

Entre as limitações deste estudo, destacaram-se a natureza retrospectiva da análise e a ausência de um grupo controle, fatores que restringem a possibilidade de estabelecer relações causais. Também se destacaram a ausência de análise sobre a abordagem terapêutica, a utilização de dados autorrelatados e a falta de informações para a caracterização mais detalhada da amostra quanto a comorbidades e fatores de risco. O estudo apresenta como ponto forte o caráter multicêntrico e se destaca pela relevância de abordar uma temática ainda pouco explorada na literatura nacional.

Concluiu-se que a população atendida nos primeiros três anos de implementação da Linha de Cuidado da Pessoa com Doença Renal Crônica de Santa Catarina em cinco centros ambulatoriais especializados era predominantemente feminina, idosa e apresentava múltiplos fatores de risco. A elevada taxa de acompanhamento ao longo de um ano e a manutenção da função renal das pessoas apontaram para a relevância do seguimento especializado na atenção às pessoas com doença renal crônica avançada. 

Ainda que não seja possível estabelecer causalidade, tais achados sugeriram que esse tipo de cuidado pode estar associado a melhores desfechos a longo prazo para as pessoas e à redução dos custos para os sistemas de saúde. Além disso, ressaltou-se a importância de fortalecer no SUS estratégias organizacionais que facilitem o acesso e a adesão ao acompanhamento especializado, como agilidade para a marcação da primeira consulta, agendamento imediato do retorno e oferta de transporte gratuito. Isso contribui para diminuir a velocidade de progressão da doença e promover maior sustentabilidade do sistema.

## Data Availability

Os dados utilizados neste estudo estão disponíveis, mediante solicitação ao autor correspondente, para pareceristas e leitores interessados.
